# 

**Published:** 1820-06-01

**Authors:** 


### Medico-Chirurgical Review,

AND

### JOURNAL

OF

### MEDICAL SCIENCE.

(Quarterly.)

EXHIBITING A COMPREHENSIVE ANALYTICAL RECORD OF PROGRESSIVE MEDICINE AND SURGERY;

*EQUALLY ADAPTED TO ALL RANKS OF THE PROFESSION.*

CONDUCTED BY
ASSOCIATED PHYSICIANS AND SURGEONS;

AND SUPERINTENDED BY

JAMES JOHNSON, M. D.

AUTHOR OF A TREATISE ON TROPICAL CLIMATES, AND ON DISEASES OF THE LIVER.

(Analytical Series.)

S. G. O. LIBRARY 20567

VOLUME I. for 1820.

Nec tibi quid liceat sed quid fecisse decebit
Occurrat mentemque domat respectus honesti. CLAUD.

London:
Printed by G. HAYDEN, Little College Street, Westminster,
FOR THE EDITOR,
No. 58, Spring Gardens,
[Where Books for Review and Critical Communications, &c. *(post paid)* are to be directed.]

Published by BURGESS & HILL, Windmill Street, Haymarket; BALDWIN, CRADDOCK, & JOY, Pater-Noster Row; HIGHLEY & SON, and T. and G. UNDERWOOD, Fleet Street; J. CALLOW, Princes Street, Soho; COX & SON, Borough, ANDERSON, West Smithfield; ADAM BLACK, Edinburgh; HODGES and M'ARTHUR, Dublin; Messrs. TREUTTLE & WURTZ, Paris and Strasburgh.

1821.

W1
ME759

Dr. Matthew Baillie, Cavendish-square.

Austin, Mr. John, Haslemere, Surrey
Bacon, Dr. Charles Edward, Guildford
Barton, Frances, Esq. Whitby
Boxwill, Surgeon, Altryleix
Bourne, Mr. Surgeon, Cheadle, Staffordshire
Brere, Dr.
Bridger, Mr. Surgeon, Hon. East India Company's Service
Carden, Arthur, Esq. Templemore
Cashill, Hemphill, Dr.
Cheyne, John, M. D. Dublin
Church, W. H. Esq. East Acton
Clark, James, M. D. Dublin
Clark, Rev. Dr. John, Durham
Cole, Dr. John, Surgeon to the Forces
Crampton, Philip, Esq. Surgeon-General, Dublin
Creighton, Mr. Andrew, R. N.
Cuming, Dr. Dublin
Derbishire, Mr. John, Cheadle, Staffordshire
Derinzy, Dr.
Douglas, J. M. D. Dublin
Eberle, Dr. John, Philadelphia, in exchange for American Medical Recorder
Evans, Surgeon, Kilkenny
Fodderick, George F. M. D. Dublin
Freer, J. Esq. Assistant Surgeon, 4th Dragoon Guards
Gibbons, John, Esq. Ballymahon
Heenan, Dr. Parsons' Town
Hunterian Society, St. Mary Axe
Johnston, Andrew, Esq. Dublin
Jones, Dr. Dundalk
Kennedy, Dr. Strathearne, Perthshire
Kenny, Dr. Castle Pollard
Kingsley, Dr. Templemore
Lawder, Surgeon
Lee, Dr. Rob. Melbourn House, Whitehall
Lloyd, Dr.
M'Alister, James, Esq. R. N.
M'Calman, Mr. Duncan, Assistant Surgeon, Bengal Medical Establishment
M'Douell, Ephraim, Esq. Dublin
Marks, Mr. W. Surgeon, Stapleton-place, near Bristol
Matthias, Mr. W. Gibraltar
Medical Association of the Col. of Physicians of Ireland
Moule, Mr. W. B. Surgeon, 60th Regiment
Neilson, Dr. Dundalk
O'Beirne, James, Esq.
O'Reilly, Richard, Esq. Dublin
Paris, Dr. John Ayrton, Fellow of the Royal College of Physicians, Dover-street, Piccadilly
Parry, Dr. Charles, Bath
Peacock, George, Esq. Longford
Probart, Mr. Hairarden
Reid, Robert, M. D. Dublin
R[illegible]mond Hospital Reading Room
Rickwood, W. Esq. Surg. Horsham
Robinson, Dr. N. J. Barry
Rogers, Mr. James, Member of the Royal College of Surgeons, Settle
Rogers, Mr. Worthing
Rukwood, W. jun. Horsham
Ryan, Dr. Ed. G. Kilkenny
Scott, Mr. William, Academical Institution, Edinburgh
Staunton, Mr. J. Longbridge
Todd, C. H. Esq. Dublin
Watson, S. Esq. Surgeon, Foot Guards
Willsher, Mr. George, Weathersfield, Essex
Wilkinson, Mr. Richard, Dudley, Staffordshire
Young, Dr.

*** What is meant by Subscriber, is merely he who takes in this Journal through his own book-seller, the same as any other periodical work There is no Subscription to pay beyond the price marked on the cover, which is paid to the Subscriber's own book-seller. Subscribers are requested to send their names before the middle of August next, for insertion in No. 2 of the Journal, to be published on the 1st of September.

